# Antimicrobial Resistance and Molecular Characterization of Methicillin-Resistant *Staphylococcus aureus* from Two Pig Farms: Longitudinal Study of LA-MRSA

**DOI:** 10.3390/antibiotics11111532

**Published:** 2022-11-02

**Authors:** Majda Golob, Mateja Pate, Darja Kušar, Urška Zajc, Bojan Papić, Matjaž Ocepek, Irena Zdovc, Jana Avberšek

**Affiliations:** Institute of Microbiology and Parasitology, Veterinary Faculty, University of Ljubljana, Gerbičeva 60, SI-1000 Ljubljana, Slovenia

**Keywords:** livestock-associated methicillin-resistant *Staphylococcus aureus* (LA-MRSA), pigs, multidrug resistance (MDR), *spa* typing, longitudinal study

## Abstract

Pigs were identified as the most important reservoir of livestock-associated methicillin-resistant *Staphylococcus aureus* (LA-MRSA), mostly belonging to the emergent zoonotic clonal complex (CC) 398. Here, we investigated the presence of MRSA in sows and piglets over a period of several months in two pig farms (intensive farm A and family-run farm B). Isolates underwent antimicrobial susceptibility testing, PCR characterization and *spa* typing. We collected 280 samples, namely 206 nasal swabs from pigs and 74 environmental samples from pig housings at 12 consecutive time points. A total of 120/161 (74.5%) and 75/119 (63.0%) samples were MRSA-positive in farms A and B, respectively. All isolates harbored *mecA* but lacked *mecC* and PVL-encoding genes. The identified *spa* types (t571, t034, t1250 and t898 in farm A, t1451 and t011 in farm B) were indicative of CC398. Antimicrobial resistance patterns (all multidrug resistant in farm A, 57.2% in farm B) depended on the farm, suggesting the impact of farm size and management practices on the prevalence and characteristics of MRSA. Due to the intermittent colonization of pigs and the high contamination of their immediate environment, MRSA status should be determined at the farm level when considering preventive measures or animal trade between farms.

## 1. Introduction

Multidrug resistance (MDR) is a growing problem worldwide and has been identified as one of the greatest threats to human health [1]. The emergence of MDR bacterial strains in farmed animals is important not only from the animal health perspective, but also in terms of food safety and public health protection [2]. Methicillin-resistant *Staphylococcus aureus* (MRSA) was initially a concern in human medicine but gained additional importance when MRSA clonal complex (CC) 398 was discovered to colonize pigs and humans in close contact with them [3,4,5]. Since its initial description, livestock-associated MRSA (LA-MRSA) CC398 has been isolated from various livestock species (horses, cattle and poultry) and is the predominant LA-MRSA lineage in Europe [2,4,6,7]. The prevalence of its nasal carriage in pigs and contamination of pig slaughter batches can be very high [8,9]. In pigs, LA-MRSA CC398 is of limited clinical importance for animals but poses a risk of zoonotic transmission not only to people in close contact with livestock or dust in intensive animal housings, but also to the community through the food chain [10,11,12]. LA-MRSA has also been introduced into the healthcare settings in areas with high density of livestock farming [13].

Over the years, antibiotic use in pig production has resulted in a subpopulation of MRSA resistant not only to methicillin, but also to several other antimicrobials [14,15,16,17], and a positive association between antibiotic use in pig farming and MRSA resistance levels has been demonstrated [18]. Increasing levels of antimicrobial resistance of LA-MRSA in pig farms, both in terms of a higher proportion of resistant isolates and a broader range of antimicrobials which are no longer effective against LA-MRSA, are concerning. Recent studies reported high levels of MDR in LA-MRSA isolates from pigs [19,20,21]. However, to date, LA-MRSA isolates from pigs remain susceptible to critically important antimicrobials (CIA) such as teicoplanin, linezolid, vancomycin, rifampicin and mupirocin [19,20].

Duration and intensity of direct contact with animals and the number of positive animals in farms have been linked with colonization and infection of humans with LA-MRSA CC398 [22,23,24,25]. Because LA-MRSA CC398 survives well in farm dust, environmental contamination could contribute to its dissemination [26]. Previous longitudinal studies have shown that pigs in a MRSA-positive herd are only transiently colonized and can change MRSA status several times during the production cycle [27]. Both pig age and farm management practices affect the colonization rates in pigs [27,28]. By investigating the impact of these two factors on the occurrence of MRSA in pig farms, more targeted control measures could be implemented to prevent MRSA transmission. Working with certain age groups of pigs has been identified as a risk factor for MRSA colonization in farm workers [29]. Most previous longitudinal studies on MRSA occurrence in pigs have not determined MRSA status at the level of individual animal and have rarely included farms of different sizes and farm management practices.

Until the present study, investigations on LA-MRSA in Slovenia were limited in number. The European Food Safety Authority (EFSA) baseline study conducted in 2008 [6,8] reported a 7% prevalence in different Slovenian pig production holdings. Another study investigated links between animals and humans in a few small livestock holdings [11]. Herein, we describe a longitudinal study on the occurrence of LA-MRSA in two pig farms: a large holding with intensive pig production and a smaller family-run farm. The aim of the study was to (i) evaluate the dynamics of MRSA colonization in both sows and pigs of different age categories over several months and (ii) assess antimicrobial susceptibility and molecular characteristics (*mecA*, *mecC* and PVL-encoding genes and *spa* type) of MRSA isolates from the studied pig farms.

## 2. Results

### 2.1. MRSA Prevalence and Molecular Characteristics

Of the 280 samples (nasal swabs from pigs and dust from the pig housings) analyzed, 195 (69.6%) were confirmed as MRSA-positive, namely 127 (61.7%) from pigs and 68 (91.9%) from their environment (Table 1). All isolates were positive for *mecA* but negative for *mecC* and PVL-encoding genes. All the identified *spa* types had similar repeat succession patterns, differing in insertions and deletions of repeats (Table 1), and are known to be associated with LA-MRSA CC398 [30].

In farm A, *spa* types t571 and t034 predominated (Table 1). *Spa* type t571 was identified in 47.5% of pig and 52.4% of environmental isolates, whereas t034 was identified in 42.5% of pig and 42.9% of environmental isolates. *Spa* type t1250 was found only in pig isolates. One environmental isolate belonged to t898. In farm B, two *spa* types were identified, namely t1451 and t011 (Table 1). *Spa* type t1451 was similarly distributed among pig (70.0%) and environmental (65.7%) isolates, as was *spa* type t011, which was found in 30.0% of pig and 34.3% of environmental isolates.

The dynamics of MRSA colonization of sows and pigs of different age groups and the results for environmental samples collected during the study period are presented in Table 2 for farm A and in Table 3 for farm B.

In both farms, most (91.9%) environmental samples tested positive for MRSA (Table 1, Table 2 and Table 3). In sows, the prevalence of MRSA colonization differed between both farms. Three out of ten sows in farm A were colonized with MRSA at sampling 1–6 days before farrowing (ante partum, a.p.), but all tested MRSA-positive at the time of farrowing or immediately after it (post partum, p.p.) (Table 2). In both farms, the sampling in the week before farrowing was performed after the sows underwent showering and were placed into cleaned and disinfected farrowing wards. In farm B, sampling was additionally performed one month before farrowing with six out of seven sows being colonized with MRSA, whereas only three sows remained MRSA-positive in the week (4 days) before farrowing and only one sow immediately after farrowing (Table 3); one sow in farm B was excluded from breeding and was sampled only before farrowing. Overall post-farrowing MRSA prevalence in sows was higher in farm A than in farm B. Namely, the prevalence was 100% (10/10) or 16.7% (1/6) immediately after farrowing (0–2 or 2 days p.p.) and 100% (10/10) or 66.7% (4/6) at the second sampling of sows after farrowing (21–26 or 38 days p.p.) in farms A and B, respectively (Table 2 and Table 3). The comparison of MRSA prevalence in sows at the second sampling should be interpreted with some caution since the sampling was not conducted at exactly the same time. However, only the piglets were relocated in farm B but the sows that remained in the same compartments were sampled. In farm B, different sows tested positive at the second sampling (38 days p.p.) than at the first sampling (2 days p.p.) after farrowing. Two months after farrowing, sows were sampled again, and their MRSA colonization rates decreased in both farms: 85.7% (six out of seven; one sow was excluded from breeding and two were not found at the time of sampling) in farm A and 33.3% (two out of six) in farm B (Table 2 and Table 3). In both farms, all sows changed their MRSA status at least once during the study period. In farm A, two out of three boars that were sampled one month after the beginning of sampling also tested positive.

In piglets, the cumulative proportion of MRSA colonization was 87.5% (35/40) in farm A and 33.3% (8/24) in farm B (Table 2 and Table 3). Piglets were colonized with MRSA immediately after farrowing, but mostly in association with colonization of sows. Namely, the prevalence of MRSA in piglets was 100% (10/10) in farm A, as also reported for the sows, and 33.3% (2/6) in farm B, where one of the MRSA-negative sows had piglets that tested positive. Later, some piglets remained positive, but others tested negative. At the second and third sampling of piglets, namely at approximately one (7–12 days p.p. in farm A and 10 days p.p. in farm B) and two (14–19 or 16 days p.p.) weeks of age, their MRSA prevalence was 70.0% (7/10) or 33.3% (2/6) and 90% (9/10) or 66.7% (4/6) in farms A and B, respectively (Table 2 and Table 3). However, the most prominent difference in MRSA colonization of piglets was observed in the third week after farrowing (21–26 days p.p. in farm A and 24 days p.p. in farm B) since almost all (9/10) piglets were MRSA-positive in farm A, comparable to the prevalence in sows, but none (0/6) in farm B, which was not consistent with the prevalence in sows when sampled 14 days later.

During the post-weaning period, high rates of MRSA colonization were observed in both farms. Namely, 71.4 % (20/28) of growers and fattening pigs were MRSA-positive in farm A and 84.6% (11/13) in farm B.

### 2.2. Antimicrobial Resistance of MRSA Isolates

**Figure 1:**
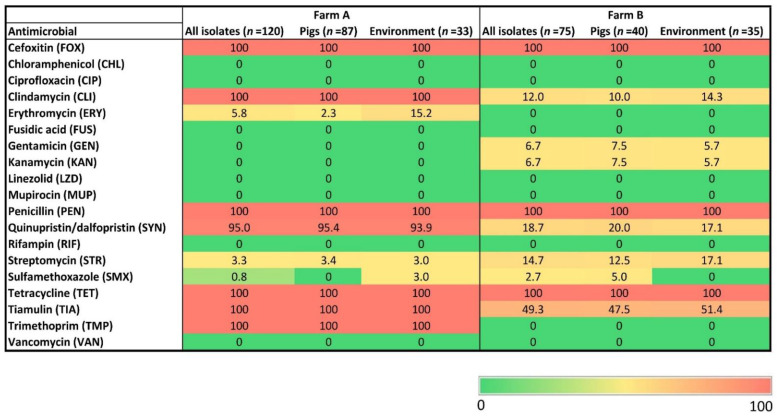
Heatmap showing the percentages of antimicrobial resistance of 195 MRSA isolates stratified by farm and isolation source. The color scale is percentile-transformed.

All studied isolates were resistant to cefoxitin, penicillin and tetracycline, but susceptible to chloramphenicol, ciprofloxacin, fusidic acid, linezolid, mupirocin, rifampin and vancomycin. Susceptibility to the other tested antimicrobials varied between the farms. The most prominent and significant differences in resistance between farms A and B were observed for clindamycin (100% vs. 12%; *p* < 0.0001), quinupristin/dalfopristin (95.0% vs. 18.7%; *p* < 0.0001), tiamulin (100% vs. 49.3%; *p* < 0.0001) and trimethoprim (100% vs. 0%; *p* < 0.0001) (Figure 1). In general, isolates of a given *spa* type did not share a common resistance pattern.

**Figure 2:**
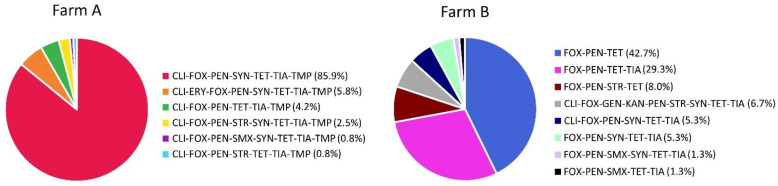
Pie chart showing the percentage of antimicrobial resistance patterns of 195 MRSA isolates on both farms.

MRSA MDR levels were high in both farms but were significantly higher in farm A than in farm B (100% vs. 57.2%; *p* < 0.0001). About one-third (37.3%) of isolates from farm B were resistant to three antimicrobial groups (β-lactams, tetracyclines and pleuromutilins or aminoglycosides), whereas all isolates from farm A were resistant to at least five antimicrobial groups (Figure 2). Six different resistance patterns were observed in farm A, whereas eight patterns were observed in farm B. The most common resistance patterns were CLI-FOX-PEN-SYN-TET-TIA-TMP in farm A and FOX-PEN-TET in farm B (Figure 2); the abbreviations of antimicrobials are explained in Figure 1. Interestingly, two boar isolates from farm A had different antimicrobial resistance patterns (the most common pattern and a less common pattern CLI-FOX-PEN-STR-SYN-TET-TIA-TMP).

## 3. Discussion

Antimicrobial resistance is one of the greatest threats to public health, and MRSA is one of the six priority pathogens responsible for deaths attributable to AMR [1]. LA-MRSA CC398 can cause the same infections as other *S. aureus*, ranging from localized skin and soft tissue infections to life-threatening bacteremia, infective endocarditis and toxic shock syndrome [4,31]. An important risk factor for LA-MRSA colonization or infection in humans is prolonged direct contact with livestock, but livestock can also acquire LA-MRSA from colonized humans [5,11,32]. The prevalence of CC398 among Slovenian human MRSA isolates increased from 15.2% in 2010 to 25.9% in 2015, ranking Slovenia third among EU countries after the Netherlands and Denmark [33,34]. Since MRSA-colonized pigs represent a risk factor for the acquisition of MRSA CC398 by humans [3,4,5], we aimed here to study the situation in the Slovenian pig industry. We investigated the MRSA colonization rates in two Slovenian pig farms with different farm management practices, which may contribute to MRSA dissemination and strain characteristics. The first notable finding was that the vast majority (91.9%) of environmental samples were MRSA-positive, which is consistent with other studies confirming dust as a potential source of MRSA colonization in both pigs and farmers or veterinarians [10,26]. It has been previously reported that LA-MRSA survives well in the farm dust with a half-life of five days and can be detected in dust for weeks, depending on the initial contamination level [26].

Here, transient MRSA carriage was observed in sows and piglets throughout the production cycle. In both farms, about one-third of the pregnant sows were colonized with MRSA in the week before farrowing, whereas immediately after farrowing, all sows in farm A tested positive. This shift could be related to the stress associated with farrowing, which can negatively impact the sow’s immune system. On the other hand, all environmental samples tested positive up to 3.5 months after farrowing in the intensive-breeding farm A, indicating high environmental contamination with MRSA and lower efficiency of animal showers or farrowing-ward disinfection. In contrast, farm B was a family-run farm with only one out of six sows being positive at farrowing and an overall lower MRSA prevalence in sows after farrowing. However, even in farm B, the environment was highly contaminated with MRSA and likely contributed to MRSA colonization of sows (other sows were positive at 38 days p.p. than at 2 days p.p.), piglets (one MRSA-negative sow had MRSA-positive piglets) as well as growers and fattening pigs (even more of them positive in farm B than in farm A). It has been previously demonstrated that MRSA status in pigs can change several times during the production cycle, which can be explained by the repeated exposure of pigs to MRSA in the contaminated farm environment [27].

The high colonization rate of piglets in farm A is not surprising since it has been reported before in farms with intensive farming systems [35]. Moreover, the MRSA status of the piglets mostly reflected that of the sows. Namely, more sows were positive after farrowing in farm A, which was reflected in a high MRSA colonization rate in the piglets, in contrast to farm B. This is consistent with previous findings which showed that pigs from MRSA-positive sows were more likely to test positive, especially before 30 days of piglet age [28]. On the contrary, if sows are MRSA-negative, pigs are more likely to change MRSA status from negative to positive after 30 days of age [28], which is consistent with the present results showing that more growers and fattening pigs were positive in farm B than in farm A. In both farms, growers and fattening pigs were positive up to three months of age with lower prevalence of MRSA in later months. This suggests that weaning is a stressful event that increases the risk of colonization, along with mixing of MRSA-positive and negative pigs, environmental contamination and handling by MRSA-colonized humans [28]. MRSA-positive fattening pigs represent a risk for meat contamination since the colonization rates at the time of slaughter are likely more relevant than colonization earlier in life, as previously shown [28]. However, the present results showed that MRSA status in some pigs changed after three months of age, which again may be attributed to intermittent colonization rather than an age-related decline in MRSA prevalence in older pigs, as shown previously [36].

Farm management is an important factor for MRSA dissemination, mainly influenced by herd size and production type, in addition to the importation of MRSA-positive animals [8,37,38,39]. MRSA transmission between pigs occurs through direct and indirect contact [35,40]. A recent study has shown that environmentally driven transmission is sufficient for MRSA to persist in the herd, without assuming the presence of persistent shedders [41]. In the present study, management practices differed between the two farms; farm A was a large pig production holding with employed workers, whereas farm B was a smaller holding where the owners were responsible for all care of the animals. It is possible that special care and attention were paid to the animals in farm B, leading to better breeding conditions and health status of the pigs as well as lower mortality. It is assumed from the farm visits that sampling, hygiene, biosecurity and sanitary measures were probably similar in both farms. Several approaches to reduce the occurrence of MRSA in pig herds have been reported. Avoiding mixing of pigs in the finishing units in herds with low LA-MRSA prevalence was shown to reduce LA-MRSA colonization [41]. Keeping pigs on straw and implementing simple cleaning reduced the occurrence of LA-MRSA during the fattening period [42]. However, knowledge gaps remain regarding the transmission and shedding of LA-MRSA in pigs of different age groups as well as on the efficacy of cleaning and disinfection measures to reduce the environmental load of LA-MRSA [41]. Therefore, further studies are needed to address this challenge.

At the time of conducting the study, about 271,000 pigs were bred in Slovenia. Since the Slovenian pig industry does not have sufficient breeding pig production of its own, it relies on the import of pigs from other European countries. Farm A imported animals from countries with a high prevalence of MRSA-positive pig herds (95% of conventional pig breeding herds in 2019 in Denmark [43]; 39–59% in 2011 in Germany [38]). Farm B imported animals from Austria, for which no recent data on MRSA colonization rates in pigs are available. This was reflected in the identified *spa* types, which belonged to the most prevalent types t034 and t011 among pigs in the mentioned countries, where *spa* types t571 and t1451 were also found but in a smaller proportion [38]. In addition, one isolate from the environment in farm A belonged to *spa* type t898, which has been only rarely reported so far [23,44]. It was reported that the number of MRSA-positive farms with breeding pigs is significantly associated with the number of pigs imported into the country [8]. Farms with MRSA-positive suppliers are 11 times more likely to become MRSA-positive than farms with MRSA-free suppliers [45]. Once MRSA is introduced into the pig population, intensive trade relations accelerate the dissemination of MRSA among herds. The presence of MRSA *spa* type t011 on the tires of the car trailer transporting pigs from farm B suggests transmission routes other than import of live animals (e.g., transport vehicles and veterinarians).

Abreu and colleagues [19] found a significant increase in resistance of MRSA from pigs to gentamicin, tobramycin, trimethoprim-sulfomethoxazole, clindamycin, fosfomycin and tigecycline between 2009 and 2018. At their first screening in 2009, 19.5% of isolates were susceptible to all antibiotics tested except to β-lactams and tetracycline. However, in the last studied period, resistance to a higher number of antibiotics was recorded in MRSA from pigs, which poses a concern for public health [46]. LA-MRSA CC398 is resistant to nearly all β-lactams and several non-β-lactam antimicrobials [47]. The resistance rates to tetracycline, β-lactams, lincosamides, trimethoprim, tiamulin, streptogramins, aminoglycosides and macrolides that we observed in farms A and B were similar to those reported in other EU countries [20,30,48].

Common antimicrobials used in pig farms are β-lactams, tetracyclines, sulfonamides, lincosamides, macrolides and quinolones [49]. Penicillins and tetracyclines are the most commonly sold antibiotics in Slovenia for the treatment of various bacterial infections in food-producing animals [50]. Antibiotic use is associated with the type and size of pig farm, with finisher and larger farms generally using more antibiotics than farrow-to-finish and smaller farms [49]. The present findings corroborate this observation, since all isolates from farm A were MDR and exhibited six distinct resistance patterns, comprising six to eight antimicrobials. The proportion of MDR isolates was significantly lower in farm B, where eight different resistance patterns comprising three to nine antimicrobials were found. Treatment protocols and antibiotic use in farm A depended on age-specific diseases and common pathogens circulating in the farm. Marbofloxacin and trimethoprim-sulfamethoxazole were usually used for individual sow treatment. Amoxicillin with clavulanic acid and aminoglycosides (gentamicin and neomycin) were given to the piglets during suckling and post-weaning periods (personal communication with the farm veterinarian). In farm B, only amoxicillin was used for the treatment of pigs of all age categories (personal communication with the farm owner). The reported use of antibiotics in the studied farms was not reflected in the resistance patterns observed, except for 100% resistance to trimethoprim in farm A. High resistance rates to trimethoprim and trimethoprim/sulfamethoxazole have already been detected in MRSA isolates from pigs [20,51,52]. Despite the reported use of gentamicin in farm A, all isolates were susceptible to this antibiotic.

Isolates from both farms were resistant to tiamulin (100% in farm A and 49.3% in farm B), which is used in veterinary medicine, especially in pigs and poultry. Recent data show resistance to tiamulin of up to 50% in MRSA from fattening pigs and up to 100% in MRSA from pork [7,21]. A notable observation was the 100% resistance rate to clindamycin in farm A. High resistance rates to clindamycin in pig isolates have been reported before [20,52]. Lopes and colleagues [18] reported a relatively high proportion (25%) of MRSA susceptible to erythromycin and resistant to clindamycin, which was mostly detected in isolates from pigs receiving no antibiotic in the feed regimen (90%). In the present study, this uncommon resistance phenotype was observed in 94% and 12% of isolates in farms A and B, respectively.

A significantly higher resistance to quinupristin/dalfopristin (SYN) was observed in farm A compared with farm B (95.0% vs. 18.7%). However, most (91.4%) isolates resistant to SYN had the minimum inhibitory concentration (MIC) values of 2 µg/mL, which is the lowest limit for resistance according to the epidemiological cut-off values (ECOFFs). If the limits proposed by the European Committee on Antimicrobial Susceptibility Testing (EUCAST; clinical cut-off value for resistance is MIC above 2 µg/mL) were considered, all these isolates could be interpreted as susceptible. Resistance to SYN was also reported for fattening pigs in Germany (32%) [38]. Recent studies on MRSA in pigs showed both low and very high resistance rates to SYN [18,52].

In this study, all isolates were susceptible to chloramphenicol, ciprofloxacin, fusidic acid, linezolid, mupirocin, rifampin and vancomycin. Other studies also reported susceptibility of MRSA to CIA in human medicine, i.e., vancomycin, teicoplanin, linezolid, daptomycin or new MRSA-susceptible cephalosporins [18,19,20,52,53]. Although LA-MRSA isolates are often MDR, several therapeutic alternatives remain available, including antimicrobials for the treatment of MRSA infections in humans.

We acknowledge certain limitations of the present study. First, the timing of sow sampling in both farms was not fully coordinated. Nevertheless, the study design allowed us to gain general insight into the dynamics of MRSA colonization in the period from 1–6 days before farrowing to six months after farrowing. Second, only one MRSA isolate per sample was investigated, resulting in partial insight into MRSA diversity in terms of antimicrobial susceptibility and *spa* types. With regard to cross-border transmission of MRSA, detailed data on importation of pigs (e.g., place of origin, time of importation, MRSA status of pigs and possible characteristics of MRSA isolates from farms of origin) would add value to the interpretation of the obtained results.

## 4. Materials and Methods

### 4.1. Study Design and Sampling

Samples were collected from pigs and the environment in two MRSA-positive pig farms between February 2015 and November 2016. Farm A was a large intensive-breeding farm consisting of 2500 sows (own breeding for the replacement gilts and weaning pigs) and approximately 20 breeding boars (8 for breeding, imported from Denmark, and 12 for fattening pigs, imported from Germany). The farm had a capacity of 70,000 growers per year. Farm B was a smaller, family-run farm with approximately 40 sows (only for fattening pigs; imported from Austria) and 1 breeding boar. The farm had a capacity of 800 fattening pigs per year.

Nasal swabs from pigs of different age categories (pig samples, *n* = 206) and dust samples from the pig housings (environmental samples, *n* = 74) were examined. Nasal samples were taken with sterile dry cotton swabs by scrubbing on the nasal mucosa of both nostrils. The sows were sampled six times on farm A and seven times on farm B: during gravidity (1–6 days before parturition and additionally one month before parturition on farm B), at the time of farrowing and the piglet weaning and three more times. Samples of ten sows were collected in farm A and samples of seven sows in farm B. The sampling schedule for the pooled samples of piglets related to the examined sows depended on the age category of animals and was planned as follows: samples from suckling piglets were collected four times (0–2, 7–12, 14–19 and 21–26 days after parturition), samples from growers three times (at ages of 35–40 days, two and three months) and samples from fattening pigs four times in farm A (at ages of 3.5, 4.5, six and nine months) and three times in farm B (at ages of four, five and six months). Dust samples were collected from surfaces in the barns using sterile wipes. The environmental wipes were collected from the horizontal surfaces of the pen separators and from areas in contact with pigs (watering place, wall, lairs, manger and steel parts). Each wipe was individually placed in a sterile plastic pot. Three environmental wipes of dust were collected at different sites in the sampled barn in each farm at every sampling. Additionally, two environmental samples from the surroundings of the barn in farm B were collected, namely dust outside the barn and the truck for pig transportation. In farm A, three samples from boars were also collected one month after the beginning of sampling. Samples were transported to the laboratory in the cooling boxes and were analyzed on the day of sampling. In total, twelve samplings were performed in each farm (Table 2 and Table 3).

### 4.2. MRSA Cultivation and Molecular Characterization

Isolation of MRSA was performed by the standard procedure according to the European Food Safety Authority (EFSA) protocol and as described before [11,54]. The presumptive MRSA colonies (one colony per sample) were identified by the matrix-assisted laser desorption/ionization time-of-flight (MALDI-TOF) mass spectrometry (Microflex LT system; Bruker Daltonics, Bremen, Germany), according to the manufacturer’s instructions.

*S. aureus* colonies grown on sheep blood agar plates were suspended into 100 µL of Tris-EDTA buffer (Sigma by Merck, Burlington, MA, USA), boiled at 95 °C for 15 min and centrifuged at 14,000× *g* for 2 min. The supernatant was used as a template for PCR without further purification. Two triplex PCR assays were used for molecular characterization of MRSA (Table 4). PCR assay 1 [55] amplified *mecA*, *nuc* and 16S rRNA genes, and PCR assay 2 [56] amplified *mecC*, *spa* (for typing) and PVL (Panton–Valentine leukocidin)-encoding genes. In brief, the 25 µL reactions contained 1 µL of template DNA, 12.5 µL of 2× Multiplex Master Mix (Qiagen, Hilden, Germany) and 0.2 µM of each primer from PCR assay 1 or 0.45 µM of *mecC*, 0.18 µM of *spa* and 1 µM of PVL-encoding gene primers from PCR assay 2. Amplification was performed with denaturation at 95 °C for 15 min, followed by 30 cycles at 94 °C for 30 s, 55 °C for 30 s (PCR assay 1) or 59 °C for 1 min (PCR assay 2) and 72 °C for 1 min, with a final elongation step at 72 °C for 10 min. Amplicons were analyzed using the QIAxcel capillary electrophoresis system (Qiagen, Germany) in combination with QIAxcel DNA High Resolution Kit (Qiagen, Germany), according to the manufacturer’s instructions. DNA isolated from the *mecA*+, *mecC*+ and *lukF-PV*+ strains, obtained from proficiency testing organized by the EU Reference Laboratory for Antimicrobial Resistance (EURL-AR), were used as PCR positive controls.

For *spa* typing, 61/120 isolates (40 from pigs and 21 from dust) from farm A were randomly selected, whereas all 75 isolates from farm B were *spa* typed. For this purpose, *spa* gene was amplified with PCR assay 2 described above [56]. Composition of the reaction mixture was changed accordingly, with the addition of PCR-grade water to the final volume, whereas the amplification conditions remained the same. Amplicons were analyzed using the QIAxcel capillary electrophoresis system (Qiagen, Germany) prior to Sanger sequencing (SEQme, Dobřiš, Czech Republic). *Spa* types were assigned using the *spa*-typing plugin of BioNumerics 7.6.3 (bioMérieux, Applied Maths NV, Martens-Latem, Belgium).

### 4.3. Antimicrobial Susceptibility Testing

Isolates were phenotypically tested for their susceptibility to 19 different antimicrobials using the broth microdilution method to determine the MIC values. A total of 195 isolates were tested with a commercially available 96-well broth microdilution plate (EUST Sensititre; Trek Diagnostic Systems, Thermo Scientific, East Grinstead, UK) following the manufacturer’s instructions and as described previously [11]. Isolates were classified as wild-type (susceptible) or non-wild-type (resistant) according to the ECOFF values proposed by EFSA and EURL-AR [15,54]. Methicillin-sensitive *S. aureus* ATCC 29213 and MRSA UN3373 (provided by EURL-AR) were used as quality control strains.

### 4.4. Statistical Analysis

Statistical analysis was performed in GraphPad Prism 8.0.2 (GraphPad Software, San Diego, CA, USA). The relationship between two groups of categorical variables (resistance rates) was assessed using Fisher’s exact test. Bonferroni correction was used to correct for multiple comparisons and an adjusted *p*-value of ≤0.05 was considered statistically significant. Statistical analysis was not performed for *spa* types and resistance patterns because no common outcomes were observed between the compared groups.

## 5. Conclusions

The present study is the first longitudinal study of LA-MRSA dynamics in Slovenian pig production holdings, which was conducted in two pig farms with different management practices. A high prevalence of MRSA was found in pigs and the environment in both farms. Transient MRSA carriage was observed in sows and piglets throughout the production cycle, which can be attributed to repeated exposure of pigs to MRSA in a highly contaminated farm environment. MDR rates were high in both farms but were significantly higher in the large intensive farm than in the family-run farm. However, the isolates were susceptible to CIA used in human medicine. The reported use of antibiotics in the studied farms was not reflected in the resistance patterns observed. The identified *spa* types, all indicative of CC398, were consistent with those reported for EU countries from which Slovenian farms import pigs. Because of the observed high contamination of the farm environment and the intermittent colonization of pigs, MRSA status should be monitored at the farm level, especially when considering restriction of live animal trade between farms with different MRSA status.

## Figures and Tables

**Table 1:** Results of cultivation and *spa* typing for 280 samples collected in two pig farms.

Farm	Samples: Positive/Collected			*Spa* Type	*Spa* Repeat Succession *
	Pigs	Environment	Total		
A	87/125 (69.6%)	33/36 (91.7%)	120/161 (74.5%)	t571 (30/61, 49.2%) t034 (26/61, 42.6%) t1250 (4/61, 6.6%) t898 (1/61, 1.6%)	08-16-02-25-02-25-34-25 08-16-02-25-02-25-34-24-25 08-16-02-25-02-25 08-16-02-25-02-25-34-34-24-25
B	40/81 (49.4%)	35/38 (92.1%)	75/119 (63.0%)	t1451 (51/75, 68.0%) t011 (24/75, 32.0%)	08-16-02-25-34-25 08-16-02-25-34-24-25

* Source: https://spa.ridom.de/spatypes.shtml (accessed on 7 September 2022).

**Table 2:** Temporal dynamics of MRSA colonization and contamination in farm A. Full squares represent MRSA-positive samples, empty squares represent MRSA-negative samples and empty cells represent samples not collected.

Farm A	1–6 d a.p.	0–2 d p.p.	7–12 d p.p.	14–19 d p.p.	21–26 d p.p.	35–40 d p.p.	2 mth p.p.	3 mth p.p.	3.5 mth p.p.	4.5 mth p.p.	6 mth p.p.	9 mth p.p.
	1	2	3	4	5	6	7	8	9	10	11	12
Sow 1	□	■			■		**					
Piglets 1		■	□	□	□							
Sow 2	■	■			■		□			□ *		□ *
Piglets 2		■	■	■	■							
Sow 3	□	■			■		■			□ *		□
Piglets 3		■	■	■	■							
Sow 4	□	■			■		#			□ *		**
Piglets 4		■	□	■	■							
Sow 5	□	■			■		■			□		□
Piglets 5		■	■	■	■							
Sow 6	□	■			■		■			□		**
Piglets 6		■	■	■	■							
Sow 7	□	■			■		■			□		**
Piglets 7		■	■	■	■							
Sow 8	□	■			■		■			■		**
Piglets 8		■	■	■	■							
Sow 9	■	■			■		■			□ *		□ *
Piglets 9		■	■	■	■							
Sow 10	■	■			■		#			□		□ *
Piglets 10		■	□	■	■							
Growers 1/fattening pigs 1						■	■	■	□	■	□	□
Growers 2/fattening pigs 2						■	■	■	□	■	□	□
Growers 3/fattening pigs 3						■	■	■	■	■	□	■
Growers 4/fattening pigs 4						■	■	■	■	□	■	■
Environment 1	■	■	■	■	■	■	■	■	□	■	□	■
Environment 2	■	■	■	■	■	■	■	■	□	■	■	■
Environment 3	■	■	■	■	■	■	■	■	■	■	■	■

*, Pooled sample where sow was not found; **, sow excluded from breeding; #, sow not found; a.p., ante partum (before parturition); p.p., post partum (after parturition). Of note, three samples from boars were also collected in farm A one month after the beginning of sampling. d, day; mth, month.

**Table 3:** Temporal dynamics of MRSA colonization and contamination in farm B. For table legend, see Table 2.

Farm B	1 mth a.p.	4 d a.p.	2 d p.p.	10 d p.p.	16 d p.p.	24 d p.p.	38 d p.p.	2 mth p.p.	3 mth p.p.	4 mth p.p.	5 mth p.p.	6 mth p.p.
	1	2	3	4	5	6	7	8	9	10	11	12
Sow 1	□	□	□				■	□			■	■
Piglets 1			□	□	■	□						
Sow 2	■	■	■				□	□			□	■
Piglets 2			■	■	□	□						
Sow 3	■	□	□				■	□			□	□
Piglets 3			□	□	■	□						
Sow 4	■	□	□				□	■			□	□
Piglets 4			■	□	□	□						
Sow 5	■	■	□				■	■			■	□
Piglets 5			□	□	■	□						
Sow 6	■	□	**									
Sow 7	■	■	□				■	□			■	□
Piglets 7			□	■	■	□						
Growers 1/fattening pigs 1							■	■	■	□	■	■
Growers 2/fattening pigs 2							■	■	■	■	□	■
Growers 3/fattening pigs 3									■			
Environment 1	■	■	■	■	■	□	■	■	■	■	■	■
Environment 2	■	■	■	■	■	■	■	■	■	■	■	□
Environment 3	■	■	■	■	■	■	■	□	■	■	■	■
Environment 4 ^§^											■	■

^§^, Outside the farm; **, sow excluded from breeding.

**Table 4:** PCR assays used for MRSA characterization.

PCR Assay	Gene	Primer	Primer Sequence (5′➝3′)	Amplicon Size (bp)	Reference
1	16S rDNA	16S-1	GTGCCAGCAGCCGCGGTAA	886	[55]
		16S-2	AGACCCGGGAACGTATTCAC		
	*mecA*	*mecA*-1	GGGATCATAGCGTCATTATTC	527	
		*mecA*-2	AACGATTGTGACACGATAGCC		
	*nuc*	*nuc*-1	TCAGCAAATGCATCACAAACAG	255	
		*nuc*-2	CGTAAATGCACTTGCTTCAGG		
2	*spa*	*spa*-1113f	TAAAGACGATCCTTCGGTGAGC	180–600	[56]
		*spa*-1514r	CAGCAGTAGTGCCGTTTGCTT		
	*lukF-PV*	*pvl*-FP	GCTGGACAAAACTTCTTGGAATAT	83	
		*pvl*-RP	GATAGGACACCAATAAATTCTGGATTG		
	*mecC*	*mecA*_LGA251_MultiFP	GAAAAAAAGGCTTAGAACGCCTC	138	
		*mecA*_LGA251_MultiRP	GAAGATCTTTTCCGTTTTCAGC		

## Data Availability

The raw data presented in this study are available upon request.
